# Fused in Sarcoma (FUS) Protein Lacking Nuclear Localization Signal (NLS) and Major RNA Binding Motifs Triggers Proteinopathy and Severe Motor Phenotype in Transgenic Mice* [Fn FN3] ^♦^

**DOI:** 10.1074/jbc.M113.492017

**Published:** 2013-07-18

**Authors:** Tatyana A. Shelkovnikova, Owen M. Peters, Alexey V. Deykin, Natalie Connor-Robson, Hannah Robinson, Alexey A. Ustyugov, Sergey O. Bachurin, Tatyana G. Ermolkevich, Igor L. Goldman, Elena R. Sadchikova, Elena A. Kovrazhkina, Veronica I. Skvortsova, Shuo-Chien Ling, Sandrine Da Cruz, Philippe A. Parone, Vladimir L. Buchman, Natalia N. Ninkina

**Affiliations:** From the ‡ School of Biosciences, Cardiff University, Museum Avenue, Cardiff CF10 3AX, Wales, United Kingdom,; the § Institute of Physiologically Active Compounds, Russian Academy of Sciences, 1 Severniy proezd, Chernogolovka 142432, Moscow Region, Russian Federation,; the ¶ Institute of Gene Biology, Russian Academy of Sciences, 34/5 Vavilov Street, Moscow 119334, Russian Federation,; the ‖ Pirogov Russian National Research Medical University, Ostrovitianov Str. 1, Moscow 117997, Russian Federation, and; the ** Ludwig Institute for Cancer Research and Department of Cellular and Molecular Medicine, University of California at San Diego, La Jolla, California 92093

**Keywords:** Amyotrophic Lateral Sclerosis (Lou Gehrig's Disease), Animal Models, Neurodegeneration, Protein Aggregation, RNA Metabolism, RNA-binding Proteins, TDP-43, Motor Neuron Disease, Proteinopathy, Transgenic Mouse

## Abstract

Dysfunction of two structurally and functionally related proteins, FUS and TAR DNA-binding protein of 43 kDa (TDP-43), implicated in crucial steps of cellular RNA metabolism can cause amyotrophic lateral sclerosis (ALS) and certain other neurodegenerative diseases. The proteins are intrinsically aggregate-prone and form non-amyloid inclusions in the affected nervous tissues, but the role of these proteinaceous aggregates in disease onset and progression is still uncertain. To address this question, we designed a variant of FUS, FUS 1–359, which is predominantly cytoplasmic, highly aggregate-prone, and lacks a region responsible for RNA recognition and binding. Expression of FUS 1–359 in neurons of transgenic mice, at a level lower than that of endogenous FUS, triggers FUSopathy associated with severe damage of motor neurons and their axons, neuroinflammatory reaction, and eventual loss of selective motor neuron populations. These pathological changes cause abrupt development of a severe motor phenotype at the age of 2.5–4.5 months and death of affected animals within several days of onset. The pattern of pathology in transgenic FUS 1–359 mice recapitulates several key features of human ALS with the dynamics of the disease progression compressed in line with shorter mouse lifespan. Our data indicate that neuronal FUS aggregation is sufficient to cause ALS-like phenotype in transgenic mice.

## Teaser

**Background:** FUS inclusions are hallmarks of certain neurodegenerative diseases.

**Results:** Expression of a highly aggregate prone FUS variant in transgenic mice causes proteinopathy and severe motor phenotype.

**Conclusion:** Aggregation of FUS is sufficient to recapitulate motor pathology typical for amyotrophic lateral sclerosis.

**Significance:** Understanding the role of protein aggregation in the development of human neurodegenerative diseases is crucial for designing efficient therapeutic approaches.

## Introduction

Multiple missense and nonsense mutations in genes encoding DNA/RNA-binding proteins FUS^6^ and TDP-43 were strongly linked with the development of ALS and related diseases, although it is still unclear how changes in the structure and/or metabolism of these proteins mediate pathology. In motor neurons of patients with *FUS* gene mutations, the encoded protein loses its normal nuclear localization and forms characteristic cytoplasmic inclusions (1, 2). Moreover, FUS-positive inclusions have been observed in neurons of some patients with sporadic ALS (3), frontotemporal lobar degeneration (4), atypical neuronal intermediate filament inclusion disease (5), basophilic inclusion body disease (6), and Unverricht-Lundborg disease (7), signifying a role for non-genetic protein modifications in the development of FUS-induced neuropathology.

However, the question of whether FUS aggregation is sufficient to cause pathological changes typical for FUSopathies or whether its altered function in RNA metabolism plays a primary role in the pathology development is still to be answered. Findings supporting the latter mechanism were reported (8), but the importance of FUS aggregation with formation of FUS positive inclusions in the affected neurons as triggers of pathological changes has never been directly addressed. This is largely caused by the apparent difficulty of separating the effects of deregulation of FUS RNA targets by overexpressed and mislocalized protein from the immediate and RNA target-independent consequences of FUS aggregation and formation of insoluble inclusions in available *in vivo* models. Furthermore, it appeared extremely hard to achieve aggregation and respective proteinopathy in models with expression of full-length FUS or FUS lacking functional NLS (9–12), indicating that an additional event(s) is probably required to trigger aggregation of these proteins. To overcome these limitations, we have designed a FUS variant that would be predominantly cytoplasmic due to the lack of NLS and would not be able to interact with RNA and thus, directly affect RNA metabolism due to the deletion of major RNA binding domains (two C-terminal RGG boxes and a zinc finger). On the other hand, this truncated FUS 1–359 protein retained an N-terminal prion-like domain (13), allowing its efficient aggregation. Moreover, because in FUS protein similar functional domains follow an inverse C- to N-terminal order to that of TDP-43, this C-terminally truncated FUS protein structurally resembled an N-terminally truncated 25-kDa product of caspase cleavage of TDP-43 that has been previously implicated in the development of neuronal pathology (14).

Here we demonstrate that expression of a relatively low level of FUS 1–359 protein in neurons of transgenic mice triggers FUSopathy and severe motor neuron pathology, recapitulating certain key features of human diseases associated with FUS aggregation and dysfunction.

## EXPERIMENTAL PROCEDURES

### 

#### 

##### Expression Plasmids and Transfection of Eukaryotic Cells

Human *FUS* fragments carrying deletions were produced by PCR amplification from full-length cDNA using designed primers, cloned into pTOPO-Blunt vector (Invitrogen), and after verification of the insert sequence, subcloned into the pEGFP-C1 vector (Clontech) downstream and in-frame with the GFP coding region. SH-SY5Y human neuroblastoma cells were maintained in Dulbecco modified Eagle's medium (Invitrogen), supplemented with 10% fetal bovine serum. For immunofluorescence, cells were grown on poly-l-lysine-coated coverslips. Cells were transfected with expression plasmids using Lipofectamine 2000 reagent (Invitrogen) according to the manufacturer's instructions. 48 h after transfection, cells were fixed with 4% paraformaldehyde, and cell nuclei were visualized with DAPI. Epifluorescent images were taken using a BX61 microscope (Olympus) and processed using the Cell-F software.

##### Production of Transgenic Mice

A fragment of human FUS 1–359 cDNA including 9 bp of 5′-UTR was cloned into Thy-1 promoter plasmid 323-pTSC21k as described previously (15). For microinjection of mouse oocytes, a gel-purified fragment obtained by digestion of the resulting plasmid DNA with NotI was used. Transgenic animals were identified by PCR analysis of DNA from ear or tail biopsies by the presence of 255-bp product (primers 5′-TCTTTGTGCAAGGCCTGGGT-3′and 5′-AGAAGCAAGACCTCTGCAGAG-3′). Two founders on C57Bl6/CBA genetic background were produced and used to establish transgenic lines F19 and F6 by several (>7) generations of backcrosses with C57Bl6J wild type mice. All animal experiments were carried out in accordance with the UK Animals (Scientific Procedures) Act 1986.

##### Gait Analysis

Animals were trained to run along a narrow passage lined with a strip of white paper before carrying out the test run. Blue and red inks were applied to detect hind and fore limb prints, respectively.

##### Antibodies

Two polyclonal antibodies highly specific to the N terminus of human FUS protein (1480) or the N terminus of mouse FUS protein (1482) were produced in rabbits using human FUS peptide 128-GSYSQQPSYGGQQ-140 or mouse FUS peptide 129-GGYGQQSGYGGQQ-141 as antigens, respectively. Both antibodies were used in 1:2000 dilution for immunohistochemistry and Western blotting. Commercial primary antibodies against the following antigens were used: C terminus of FUS protein (mouse monoclonal, clone 4H11, Santa Cruz Biotechnology); N terminus of FUS protein (rabbit polyclonal, Abcam; mouse polyclonal, BD Biosciences); GFAP (rabbit polyclonal, Sigma); NeuN (mouse monoclonal, clone MAB377, Chemicon); ubiquitin (mouse monoclonal, clone N-19, Santa Cruz Biotechnology); neurofilament M (mouse monoclonal, clones NL6 or NN18, Sigma); synaptophysin (mouse monoclonal, clone 2, BD Transduction Laboratories); and β-actin (mouse polyclonal, Sigma). All primary antibodies were used in 1:1000 dilution for all applications.

##### Western Blotting

Cells or tissues were homogenized directly in SDS-PAGE loading buffer and denatured at 100 °C for 10 min. Equal amounts of total protein were run on SDS-PAGE and transferred to PVDF membrane by semidry blotting followed by blocking, incubation with primary and HRP-conjugated secondary (GE Healthcare) antibodies, and ECL detection. Equal loading was confirmed by reprobing membranes with antibodies against β-actin or/and GAPDH.

##### Histology, Immunohistochemistry, and Stereological Counts

Mouse tissues were fixed and embedded in paraffin wax, and 8-μm-thick sections mounted on poly-l-lysine-coated slides (Thermo Scientific) were Nissl- or hematoxylin and eosin-stained as described previously (15, 16). Immunostaining was performed using Elite plus kits (Vector laboratories) and 3,3′-diaminobenzidine (Sigma) as a substrate. For microglia detection, sections were incubated with biotinylated *Ricinus communis* agglutinin I (Vector Laboratories). For double immunofluorescence, secondary Alexa Fluor-conjugated antibodies (1:1000, Molecular Probes, Invitrogen) were used. In experiments with proteinase K treatment, the enzyme (Fermentas) was diluted in Tris buffer (10 mm Tris-HCl, pH 7.5; 5 mm EDTA) to the final concentration of 200 μg/ml. Sections were deparaffinized, rehydrated, washed with PBS, and incubated in proteinase K solution for 1 h at 37 °C in a humidified chamber. After several washes, anti-FUS staining and DAPI staining were performed. The same microscope, camera, and software were used as described above for epifluorescence imaging.

##### Neuromuscular Junctions Staining

For assessment of neuromuscular junction innervation, 4-month-old mice were transcardially perfused with 4% paraformaldehyde in PBS, and whole gastrocnemius muscles were dissected. Muscles were immediately passed through a gradient of 5–20% sucrose, cryoprotected overnight in 20% sucrose (4 °C), and embedded in CryoMount (Thermo Scientific). 35-μm-thick frozen sections were permeabilized in 5% BSA in PBS, 0.5% Triton X-100 at 37.5 °C for 4 h, incubated in primary antibodies against neurofilament M and synaptophysin in 1% BSA in PBS, 0.5% Triton X-100 overnight (room temperature), washed, and incubated with Alexa Fluor 488 secondary antibody and Alexa Fluor 648-conjugated α-bungarotoxin for 2 h at room temperature. Images were taken using a laser scanning confocal DM6000B microscope (Leica Microsystems) and processed using the Leica confocal software.

##### Semithin Sections of Sciatic Nerve and Spinal Roots

Sciatic nerves and spinal nerve roots were dissected and submerged overnight in cold 2% paraformaldehyde and 2% glutaraldehyde in 0.1 m Sorenson's phosphate buffer, post-fixed in 1% osmium tetroxide for 2 h, and embedded in araldite (Agar Scientific) at 60 °C. Semithin (0.6-μm) sections were mounted on poly-l-lysine-coated slides and stained with toluidine blue as described previously (17).

##### Cell and Fiber Counts

To assess the number of motor neurons in spinal ventral horns and in brainstem nuclei, neuronal nucleoli were counted in both ventral horns or for left and right nuclei, on every 10th Nissl-stained section, and stereological technique was used to obtain final counts per 1 mm of spinal cord length or per nucleus. All myelinated fibers were counted in six randomly chosen 0.01-mm^2^ squares on sciatic nerve sections per animal, and mean was used for statistics. The individual that performed the counts was blinded to the genotype of experimental animals.

## RESULTS

### 

#### 

##### Severe, Fast Progressing Motor Dysfunction and Early Lethality of FUS Transgenic Mice

**FIGURE 1.**
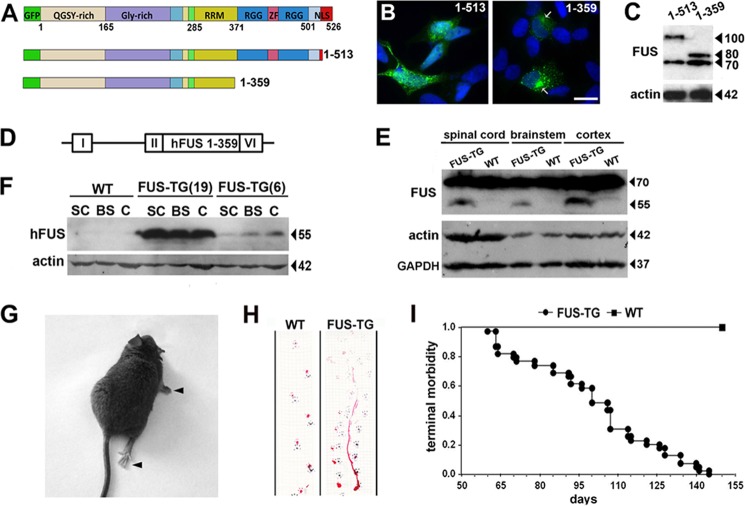
**Motor dysfunction and decreased life span in transgenic mice expressing truncated human FUS 1–359 protein.** *A*, schematic representation of FUS protein and its GFP-tagged truncated variants, FUS 1–513 and FUS 1–359, used for studies in cultured cells. *B*, typical patterns of FUS 1–513 and FUS 1–359 aggregation in neuroblastoma SH-SY5Y cells 48 h after transfection. *Arrows* point to large perinuclear inclusions formed by FUS 1–359. *C*, FUS 1–513 (∼100 kDa) and FUS 1–359 (∼80 kDa) variants are expressed at levels similar to each other and to similar to endogenous protein (∼70-kDa band) in neuroblastoma cells. *D*, map of the genetic construct used to produce transgenic animals. A cDNA fragment encoding amino acids 1–359 of human FUS (*hFUS*) was inserted into the Thy-1 expression cassette. *E*, human FUS 1–359 protein was detected in the neural tissues of transgenic (line 19) but not wild type mice using an antibody against the N terminus of FUS that recognizes both ∼55-kDa human and ∼70-kDa endogenous mouse proteins. *F*, significantly lower levels of truncated human FUS 1–359 protein in the spinal cord (*SC*), brainstem (*BS*), and cortex (*C*) of 9-month-old homozygous mice of line 6 when compared with 3-month-old hemizygous mice of line 19 were detected using antibody 1480 that specifically recognizes only human FUS. *G*, a transgenic mouse with paralyzed right forelimbs and hind limbs. See also supplemental Video S1. *H*, footprint analysis of the gait of a 4-month-old FUS 1–359 TG 19 transgenic mouse (*FUS-TG*) with paralyzed right forelimb when compared with wild type animal of the same age (*WT*). *I*, Kaplan-Meier plot of wild type (*n* = 39) and line 19 FUS-TG (*n* = 34) mice survival over a 5-month period. *Scale bar* in *B*, 15 μm.

**FIGURE 2.**
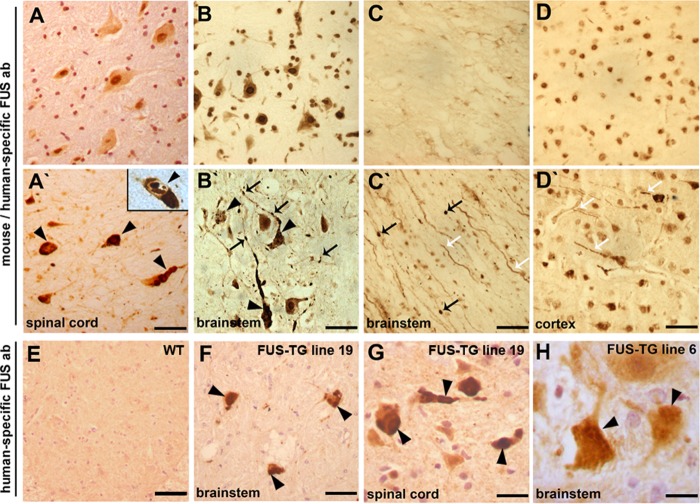
**Proteinopathy in the nervous system of FUS-TG mice.** *A–H*, paraffin sections were stained with an antibody (*ab*) recognizing an N-terminal epitope in both mouse and human FUS (*A–D*, *A′–D′*) or antibody 1480 specific to human protein (*E–H*). Multiple intracellular FUS-positive inclusions were revealed in the cytoplasm (*arrowheads*), nucleus (*inset*), and axons (*arrows*) of spinal cord (*A′*) and brainstem (*B′*) neurons of symptomatic FUS-TG but not of non-transgenic littermate (*A* and *B*, respectively) mice. Similar inclusions were detected in the brainstem (*F*) and spinal cord (*G*) of transgenic mice using human FUS-specific antibody. Truncated FUS accumulates in axons (*white arrows*) and forms spheroids (*black arrows*) in brainstem tracts of symptomatic FUS-TG (*C′*) but not of non-transgenic littermate (*C*) mice. In cortical neurons of symptomatic FUS-TG mice (*D′*), FUS accumulates and forms inclusions in cell bodies (*black arrows*) and neurites (*white arrows*), whereas in non-transgenic littermates, endogenous FUS is confined to cell nuclei (*D*). *E*, staining of a spinal cord section from a non-transgenic animal shows that human FUS-specific antibody 1480 does not react with mouse FUS protein. *H*, FUS-positive inclusions (*arrowheads*) are occasionally detected in neurons of 9-month-old transgenic mice of low-expressing line 6. *Scale bars*: *A–F*, 50 μm; *G*, 30 μm; *H*, 15 μm.

To confirm a high aggregation propensity of the engineered FUS 1–359 protein (Fig. 1*A*) in the cytoplasm of eukaryotic cells, we expressed it as a GFP fusion in the human neuroblastoma SH-SY5Y cell line. Indeed, in contrast to FUS lacking epitopes 2 and 3 of NLS (18) but retaining all other domains (FUS 1–513), which was diffusely distributed or occasionally formed small cytoplasmic granules even 48 h after transfection, FUS 1–359 rapidly aggregated and formed large perinuclear inclusions (Fig. 1*B*, *arrows*) as early as 12 h after transfection despite the same levels of expression for both proteins (Fig. 1*C*). Therefore, we proceeded to generation and characterization of transgenic mice expressing human FUS 1–359 protein in their nervous system under control of the Thy-1 promoter (Fig. 1*D*). Transgenic lines FUS 1–359 TG 6 and FUS 1–359 TG 19 were established from two independent founders. In the central nervous system of hemizygous FUS 1–359 TG 19 mice, the 55-kDa truncated human protein was expressed at a lower level than the 70-kDa endogenous mouse FUS protein (Fig. 1*E*), and these animals developed a severe neurological phenotype early in their life. Animals of FUS 1–359 TG line 6 expressed substantially less human protein in their nervous system when compared with line 19 (Fig. 1*F*), and only in aging homozygous line 6 mice were FUS-positive aggregates observed in a small proportion of lower motor neurons (see Fig. 2*H*). Therefore, we focused our studies on characterizing pathology in hemizygous FUS 1–359 TG 19 mice (herein “FUS-TG”). These mice were indistinguishable from their wild type littermates until the age of 2.5–4.5 months, at which point transgenic animals abruptly developed severe motor dysfunction. A typical pattern at the onset of clinical signs included gait impairment caused by asymmetrical pareses and eventual complete paralyzes of limbs (Fig. 1, *G* and *H*, and supplemental Video S1). The disease rapidly progressed and spread to other groups of muscles, and typically affected animals became emaciated and dehydrated and the lost righting reflex and the ability to move freely within several days of onset. Because transformation of a visibly healthy animal into one exhibiting severe pathological phenotypes starts any time within an ∼2-month interval and always occurs extremely quickly, it was not possible to obtain statistically sound data for any parameters of the disease progression for a cohort of transgenic mice. However, it was possible to predict with absolute confidence that after an individual animal develops the first signs of unstable gait or/and displays slightly compromised performance in the inverted grid test, it will reach the terminal stage of the disease within a maximum of 2 weeks. The lifespan of FUS-TG mice did not exceed 5 months (Fig. 1*I*).

##### Proteinopathy in the Nervous System of FUS Transgenic Mice

**FIGURE 3.**
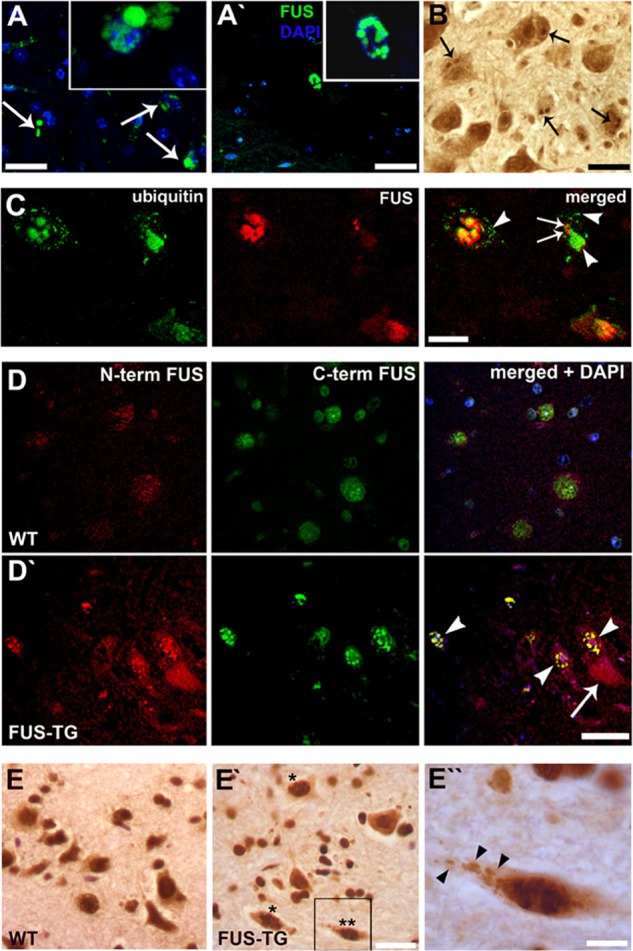
**Characterization of proteinaceous inclusions in FUS-TG mice.** *A*, FUS-positive inclusions in motor neurons of symptomatic FUS-TG mice are resistant to treatment with 200 μg/ml of proteinase K for 30 min (*A*) or 1 h (*A′*). *B*, ubiquitinated inclusions (*arrows*) in the spinal cord of a symptomatic FUS-TG animal. *C*, immunofluorescent detection of ubiquitin (*green*) and FUS (*red*) in a section through the ventral horns of the spinal cord of a symptomatic FUS-TG mouse. Some FUS-positive inclusions in motor neurons are also ubiquitin-positive, whereas other cells bear FUS-positive/ubiquitin-negative or/and FUS-negative/ubiquitin-positive inclusions (*arrows* and *arrowheads*, respectively). *D*, spinal cord sections of wild type (*D*) and FUS-TG (*D′*) mice stained with antibodies against the C terminus (*C term*) of FUS for detection of the endogenous protein (*green*) and against the N terminus (*N term*) of FUS for detection of both endogenous and human FUS 1–359 proteins (*red*). Endogenous FUS is recruited into nuclear aggregates, formed by human FUS 1–359 (*arrowheads*). A cytoplasmic FUS 1–359 inclusion (*arrow*) is negative for endogenous FUS. *E*, antibody 1482 that specifically recognizes only mouse FUS in both wild type and transgenic animals also detects intranuclear (cell marked by *one asterisk*) or even cytoplasmic (cell marked by *two asterisks* and enlarged in *E″*, *arrows*) inclusions in a subset of spinal motor neurons of FUS-TG mice (*E′*). *Scale bars*: *A* and *A′*, 50 μm; *B–E′*, 30 μm; *E″*, 10 μm.

Immunohistochemical analysis of the central nervous system of symptomatic FUS-TG mice using an antibody that recognizes the N-terminal epitope in both mouse and human proteins (Fig. 2, *A′–D′*) or an antibody specific to the human protein (Fig. 2, *F* and *G*) revealed multiple FUS-positive inclusions primarily in the lower motor neuron cell bodies, axons and, in a subset of cells, in the nucleus (Fig. 2*A′*, *inset*). Similar inclusions were observed in other neurons throughout the nervous system of symptomatic animals, including upper motor neurons in the motor cortex (Fig. 2*D′*). In the spinal cord of FUS-TG mice, large eosinophilic inclusions were occasionally detected, which were not seen in non-transgenic littermates (data not shown). Similar to proteinaceous inclusions typical for certain neurodegenerative diseases (19, 20), both cytoplasmic and nuclear inclusions in neurons of FUS-TG mice were resistant to proteinase K treatment (Fig. 3, *A* and A*′*). However, they were not stained by the amyloid-detecting dyes Congo Red or thioflavin S, which is also a feature of FUS-positive inclusions in ALS and related diseases (21). Consistent with the presence of ubiquitinated FUS-positive inclusions in these human diseases (14), ubiquitin-positive inclusions of various size and morphology were often observed in motor neurons of FUS-TG mice (Fig. 3*B*). However, double immunofluorescent staining showed that only a fraction of FUS-positive inclusions was ubiquitinated, and in some cells, non-overlapping FUS and ubiquitin inclusions were present (Fig. 3*C*). In some neurons that developed intranuclear FUS inclusions, endogenous protein (detected using an antibody that recognizes the C terminus of FUS) became recruited to these structures (Fig. 3*D′*). Immunostaining with a mouse-specific antibody also revealed nuclear and rarely cytoplasmic (Fig. 3*E′* and enlarged in Fig. 3E*″*) inclusions in spinal motor neurons. Therefore, the truncated form of FUS is able to seed aggregation of normal FUS protein.

##### Loss of Spinal Motor Neurons and Peripheral Nerve Fibers in FUS Transgenic Mice

**FIGURE 4.**
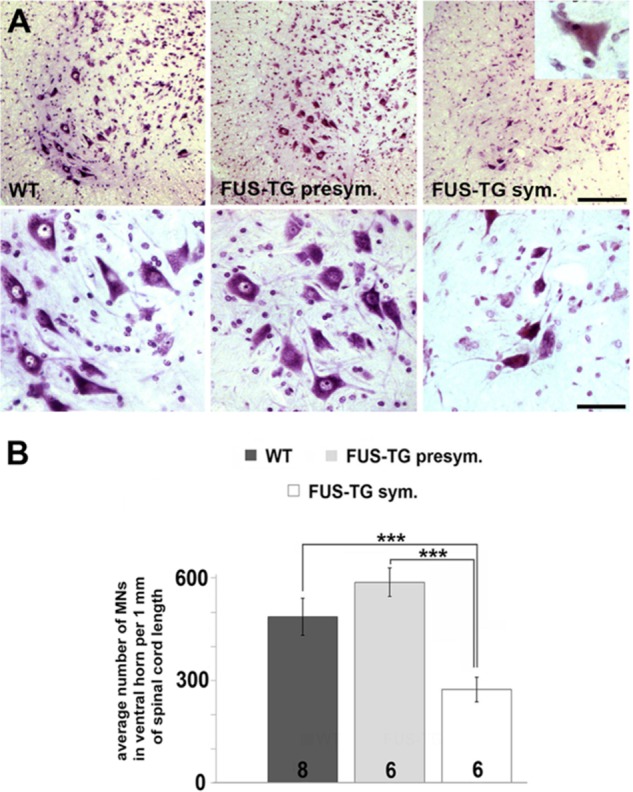
**Degeneration and loss of spinal motor neurons in symptomatic FUS-TG mice.** *A* and *B*, representative images of cresyl fast violet-stained sections through ventral spinal cord of presymptomatic (*presym.*) and symptomatic (*sym.*) FUS-TG and wild type animals (*A*) and a bar chart showing mean ± S.E. of motor neuron (*MN*) number in the ventral horn of the spinal cord (*B*). *Inset* in *A* shows degenerative changes (shrinking, chromatolysis) in a surviving motor neuron. The number of animals analyzed for the group is shown at the *bottom* of each bar (***, *p* < 0.001, Mann-Whitney test). *Scale bars*: *A* (*top panel*), 100 μm; *A* (*bottom panel*), 30 μm.

**FIGURE 5.**
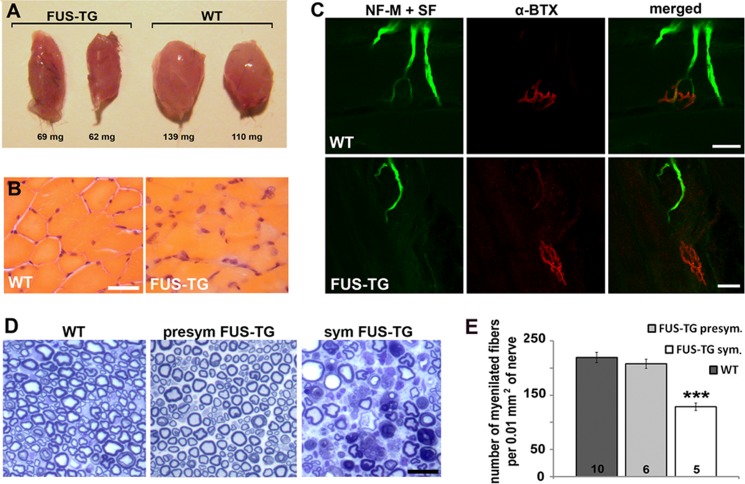
**Muscle atrophy, denervation and axonal damage in symptomatic FUS-TG mice.** *A*, prominent atrophy of skeletal muscles at terminal stages of the disease in FUS-TG mice: left and right hind limb gastrocnemius muscles dissected from a transgenic animal with hind limb paralysis when compared with those from a non-transgenic littermate control. *B*, damaged muscle fibers in gastrocnemius muscle from a paralyzed limb of a FUS-TG animal when compared with normal fibers in gastrocnemius muscle of wild type mouse (hematoxylin and eosin staining). *C*, representative confocal microscope images of the neuromuscular junctions in gastrocnemius muscles of symptomatic FUS-TG and wild type mice. Endplates were visualized by staining with α-bungarotoxin (α*-BTX*), and axons and presynapses were visualized with a combination of antibodies against neurofilament-M (*NF-M*) and synaptophysin (*SF*). *D*, representative semithin sections through sciatic nerve of symptomatic (*sym*) and presymptomatic (*presym*) FUS-TG and wild type mice stained with toluidine blue. *E*, reduced total numbers of myelinated fibers in the sciatic nerve of symptomatic but not presymptomatic animals. Bar chart shows mean ± S.E. of fiber number (***, *p* < 0.001, Mann-Whitney test); the number of nerves analyzed for the group is shown at the *bottom* of each bar. *Scale bars*: *B*, 50 μm; *C–E*, 30 μm.

Aggregation and deposition of FUS protein are accompanied by severe damage to neurons and their axons. At the symptomatic stage, FUS-TG mice lose approximately half of their spinal motor neurons, whereas the majority of remaining neurons appear shrunken and/or chromatolytic (Fig. 4, *A* and *B*). However, the most probable cause of rapidly developing muscle paralysis and muscle atrophy (Fig. 5, *A* and *B*) in FUS-TG mice is the disconnection of the motor neuron axons from the muscle endplates (Fig. 5*C*) because significant neuronal loss and damage of myelinated fibers in peripheral nerves can be observed only at the late, generalized stage of the disease (Fig. 5, *D* and *E*).

##### Selective Loss of Lower Motor Neuron Populations in the Brainstem of FUS Transgenic Mice

**FIGURE 6.**
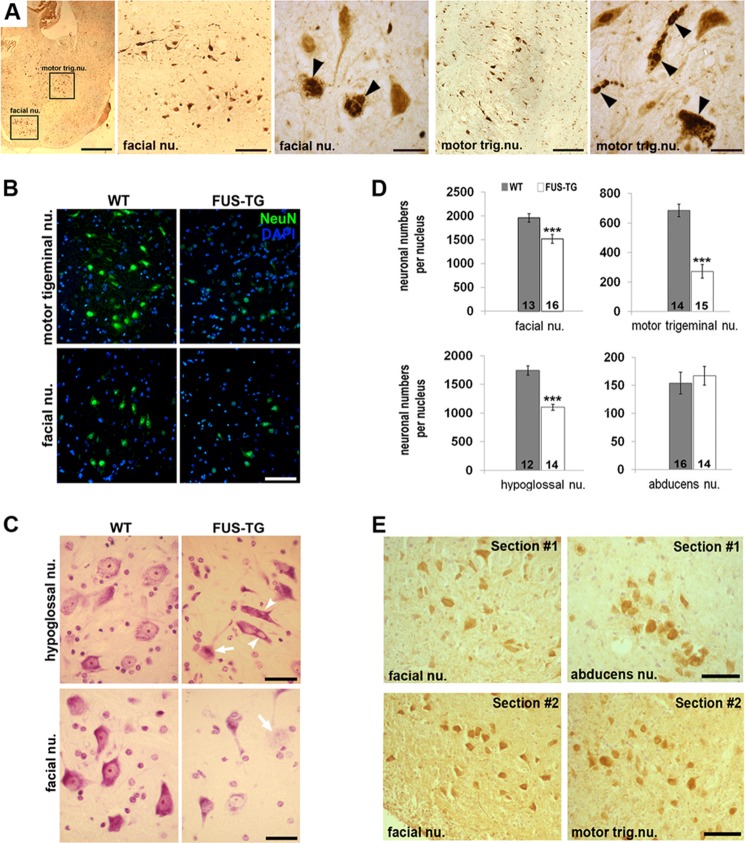
**Cranial motor neuron populations are selectively affected in symptomatic FUS-TG mice.** *A*, low and high magnification images showing accumulation of exogenous FUS and the presence of multiple FUS-positive inclusions (*arrowheads*) in facial nuclei (*facial nu.*) and motor trigeminal nuclei (*motor trig. nu.*) revealed by anti-FUS staining of a sagittal section through the brainstem of a symptomatic transgenic mouse. *B–D*, motor neurons are lost and damaged in select brainstem nuclei of symptomatic FUS transgenic mice. *B*, representative images show neurons in brainstem motor nuclei visualized using neuronal nuclear marker NeuN (*green*). *C*, surviving motor neurons in Nissl-stained sections through hypoglossal and facial nuclei appear shrunken and/or chromatolytic (*arrow*) and sometimes present with vacuolization of cytoplasm (*arrowheads*). *D*, bar charts show mean ± S.E. of motor neuron number stereologically counted in motor trigeminal, facial, hypoglossal, and abducens nuclei of severely affected FUS-TG and non-transgenic littermate mice. The number of motor nuclei analyzed for the group is shown at the *bottom* of each bar (***, *p* < 0.001, Mann-Whitney test). *E*, at the presymptomatic stage, levels of FUS 1–359 protein are similar in motor neurons of facial, abducens, and motor trigeminal nuclei. Histological sections through both facial and abducens (*Section #1*) or both facial and motor trigeminal (*Section #2*) nuclei of a presymptomatic FUS-TG mouse were immunostained with human FUS-specific antibody 1480. *Scale bars*: *A*, 250 μm for general plane image, 50 μm for images of entire nuclei, and 15 μm for close-up images; *B*, 75 μm; *C*, 20 μm; *E*, 50 μm.

Significant neuronal loss and pathological changes in remaining lower motor neurons were also observed in the brainstem motor nuclei. However, this effect was selective for certain neuronal populations. Motor trigeminal, hypoglossal, and facial nuclei were affected, whereas neurons in the nucleus abducens remained intact (Fig. 6, *A–D*), although similar levels of truncated human FUS protein expression were found in these neuronal populations at the stage preceding development of pathological changes (Fig. 6*E*).

##### Neuroinflammation in the Spinal Cord and Brainstem of FUS Transgenic Mice

**FIGURE 7.**
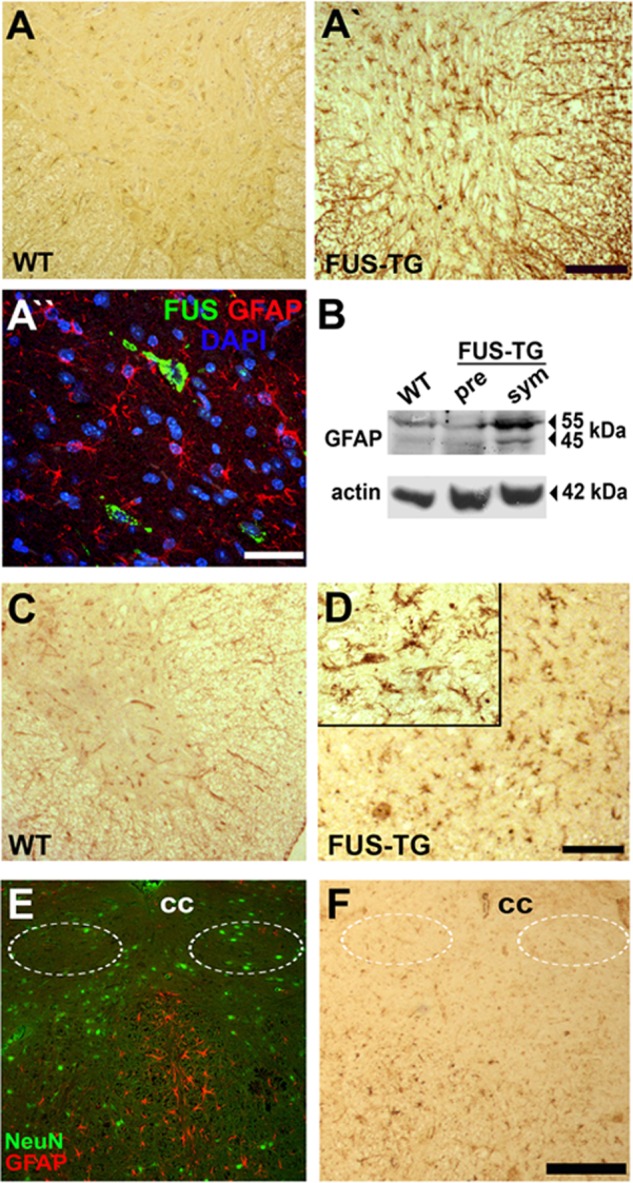
**Neuroinflammation in the nervous system of symptomatic FUS-TG mice.** *A*, immunostaining of histological sections for GFAP detects prominent reactive astrogliosis in the spinal cord of symptomatic FUS-TG mice (A*′* and A*″*) but not their littermate controls (*A*). *B*, elevated levels of GFAP protein in FUS-TG animals at the symptomatic (*sym*) stage revealed by Western blotting. *pre*, presymptomatic. *C* and *D*, prominent microgliosis detected with *R. communis* agglutinin I (RCAI) in the spinal cord of FUS-TG mice (*C*) but not non-transgenic littermates (*D*). *E* and *F*, limited astro- and microgliosis in the brainstem nuclei of FUS-TG mice. Shown is an example of two adjacent sections through the brainstem of a symptomatic FUS-TG mouse at the level of the hypoglossal nuclei (*encircled*) double immunostained for NeuN, a neuronal nuclear marker, and GFAP (*E*) or stained with biotinylated *R. communis* agglutinin I (*F*). Note that there are very few activated microglial cells and virtually no activated astroglial cells within the boundaries of the nucleus, whereas multiple positively stained cells are abundant in neighboring brain regions. The size of proteins in kDa is shown in *panel B. Scale bars*: *A*, A*′*, *C* and *D*, 100 μm; A*″*, 50 μm; *E* and *F*, 75 μm.

Profound neuroinflammation was detected in the spinal cord of FUS-TG mice using specific markers of reactive astro- and microglia (Fig. 7, *A–D*). In contrast, only limited astro- and microgliosis were detected even in the affected brainstem nuclei of symptomatic FUS-TG mice despite strong neuroinflammatory reaction in the adjacent brain structures (Fig. 7, *E* and *F*). No signs of neuroinflammation were observed in any parts of the nervous system of non-transgenic littermates (Fig. 7, *A* and *C*, and data not shown).

## DISCUSSION

We produced a transgenic mouse model that has enabled us to address the effects of *in vivo* FUS aggregation independently of its roles in cellular RNA metabolism. Possible direct effects of exogenous FUS at the RNA-related pathways were eliminated by employing a protein with deletion of essential RNA binding domains along with the NLS. The intact N-terminal region that includes the presumptive prion-like domain (13) enabled rapid aggregation of exogenous truncated protein and even engagement of endogenous mouse FUS into inclusions, suggesting that pathologically modified forms of FUS are able to seed aggregation of other, including structurally normal, FUS proteins. It is feasible that a similar mechanism might be involved in the formation of inclusions and even spreading of pathology in human FUSopathies. Moreover, sequestering of endogenous FUS in inclusions might cause depletion of its functional soluble forms in affected neurons and consequent malfunction of RNA metabolism. Such secondary loss of function along with direct toxicity of products of FUS aggregation might play a role in the development of neurodegenerative changes in the nervous system of FUS transgenic mice and patients with FUSopathy.

An abrupt development of prominent FUSopathy in hemizygous line 19 transgenic mice expressing FUS 1–359 at a level lower than the level of endogenous protein led to severe damage of myelinated axons in the peripheral nerves, loss of spinal motor neurons, and consequently, muscle paralysis and atrophy. This resulted in the death of young animals several days after the first signs of pathology, which resembles the clinical pattern of typical ALS if an average mouse lifespan is extrapolated to the human one.

A selective loss of brainstem motor neurons in line 19 mice also emulates the pattern of brainstem motor nuclei damage typical for ALS where motor neuron populations involved in eye movements remain spared. Therefore, FUS aggregation mediated by its N terminus is sufficient to cause degeneration and death of motor neurons in a very selective fashion. Surprisingly, we did not observe neuroinflammatory reaction in brainstem motor nuclei affected by the neurodegenerative process, whereas both microgliosis and astrogliosis were evident in anterior horns of the spinal cord of line 19 transgenic mice. Further studies are required to explain this phenomenon.

We also produced a second line of transgenic mice expressing FUS 1–359 in their nervous system. Consistent with the significantly lower level of transgene expression, when compared with the first line, these mice start to develop neuronal FUS-positive inclusions (Fig. 2*H*) and compromised performance in motor tests (data not shown) only at the age of 9 months. Further studies of older line 6 animals will require us to establish whether their phenotype recapitulates certain features of late onset and slow developing ALS or other FUS-related neurodegenerative diseases.

Animal models available so far could not be used to discriminate between the effects exerted by RNA binding-competent FUS proteins directly at the cellular RNA metabolism and effects caused by their aggregation and deposition in the cytoplasm. Moreover, in these models, either efficient FUS aggregation and inclusion formation have not been achieved or/and only certain aspects of ALS FUS were recapitulated. Overexpression of FUS in *Drosophila melanogaster* (9, 22) and *Caenorhabditis elegans* (11), although confirming toxic gain of function for mutant variants, did not result in the formation of FUS-positive inclusions. In a previously described line of FUS transgenic mice, the increase of human wild type FUS expression by breeding animals to transgene homozygosity was required for the development of neurological phenotype (23). In a rat model, transgenic expression of an ALS-associated variant, FUS R521C, although leading to paralysis due to muscle denervation, was not sufficient to cause proteinopathy and motor neuron loss in the spinal cord (12). Similarly, expression of FUS R521C or wild type FUS in the nervous system of mice via recombinant AAV1-driven somatic brain transfer technique did not cause formation of inclusions (24). However, in the same experimental system, a truncated FUS variant lacking both NLS and a portion of RGG3 aggregated and formed inclusions in the brain neurons, although these mice did not develop a motor phenotype or neurodegeneration at the age of 3 months (24). It is not clear whether the latter was due to the nature of aggregates formed by the FUS variant used or less efficient expression of the ectopic protein in the most sensitive neuronal populations, *i.e.* motor neurons. Unexpectedly, nuclear localization and aggregation of FUS variants completely lacking C-terminal NLS were observed in a significant number of mouse neurons in the aforementioned study (24) and in our study, and the reason for this is not clear. However, it should be noticed that the majority of data about the importance of certain motifs within the FUS molecule for nuclear import and export have been obtained in cultured non-neuronal cells. These processes might be regulated differently in various cell populations *in vivo*.

The requirement of additional factors for triggering irreversible aggregation of FUS protein *in vivo*, for example compromised binding to target RNA coupled with cell stress, might explain difficulties with establishing adequate models of FUSopathy in short-lived laboratory animals. In our model, we managed to bypass this requirement by using a highly aggregate-prone protein lacking RNA binding capacity. The phenotype in our transgenic mice reproduces key features of human ALS FUS: *i.e.* (i) the presence of FUS-positive inclusions; (ii) abrupt disease onset and fast progression; (iii) motor deficits including asymmetrical limb paralysis; (iv) muscle wasting and denervation; and (v) loss of spinal motor neurons and selective involvement of brainstem nuclei. Importantly, and unlike other available models, our FUS transgenic mice allowed us to distinguish between direct effects on the cellular RNA metabolism exerted by RNA binding-competent proteins and effects triggered solely by FUS aggregation.

In conclusion, our studies produced the first direct *in vivo* evidence that aggregation of FUS protein can *per se* trigger FUSopathy with severe damage to susceptible neurons. This suggests that protein aggregation might be considered as a promising target for therapeutic intervention in FUSopathies.

## Footnotes

6 The abbreviations used are:

FUS: fused in sarcoma
TDP-43: TAR DNA-binding protein of 43 kDa
ALS: amyotrophic lateral sclerosis
NLS: nuclear localization signal
GFAP: glial fibrillary acidic protein
TG: transgenic.
